# Heaven

**Published:** 1864-01

**Authors:** N. A. W. Priest


### HEAVEN.

BY MISS N. A. W. PRIEST.

Beyond these chilling winds and gloomy skies,
  Beyond death's cloudy portal.
There is a land where beauty never dies
  And love becomes immortal:

A land whose light is never dimmed by shade,
  Whose fields are ever vernal;
Where nothing beautiful can ever fade,
  But blooms for aye, eternal.

We may not know how sweet its balmy air,
  How bright and fair its flowers;
We may not hear the songs that echo there
  Through those enchanted bowers.

The city's shining towers we may not see
  With our dim earthly vision:
For Death, the silent warder, keeps the key
  That opes those gates elysian.

But sometimes, when adown the western sky
  The fiery sunset lingers,
Its golden gates swing inward noiselessly,
  Unlocked by unseen fingers.

And while they stand a moment half-ajar,
  Gleams from the inner glory
Stream brightly through the azure vault afar,
  And half reveal the story.

O land unknown! O land of love divine!
  Father, all-wise, eternal,
Guide, guide these wandering way-worn feet of mine
  Into those pastures vernal.

